# Clinical significance of ^18^F-FDG-PET/CT for detection of incidental pre-malignant and malignant colonic lesions: correlation with colonoscopic and histopathological results

**DOI:** 10.1007/s00432-024-05806-2

**Published:** 2024-05-20

**Authors:** Yingying Zhang, Jiangqin Han, Junpeng Li, Jinming Cao, Yeye Zhou, Shengming Deng, Bin Zhang, Yi Yang

**Affiliations:** Department of Nuclear Medicine, The First Affiliated Hospital of Soochow University, Suzhou, 215006 China

**Keywords:** PET/CT, Incidental FDG uptake, Colonoscopy, Colorectal cancer

## Abstract

**Background:**

Incidental colorectal fluorodeoxyglucose (FDG) uptake, observed during positron emission tomography/computed tomography (PET/CT) scans, attracts particular attention due to its potential to represent both benign and pre-malignant/malignant lesions. Early detection and excision of these lesions are crucial for preventing cancer development and reducing mortality. This research aims to evaluate the correlation between incidental colorectal FDG uptake on PET/CT with colonoscopic and histopathological results.

**Methods:**

Retrospective analysis was performed on data from all patients who underwent PET/CT between December 2019 and December 2023 in our hospital. The study included 79 patients with incidental colonic FDG uptake who underwent endoscopy. Patient characteristics, imaging parameters, and the corresponding colonoscopy and histopathological results were studied. A comparative analysis was performed among the findings from each of these modalities. The optimal cut-off value of SUVmax for ^18^F-FDG PET/CT diagnosis of premalignant and malignant lesions was determined by receiver operating characteristic (ROC) curves. The area under the curve (AUC) of SUVmax and the combined parameters of SUVmax and colonic wall thickening (CWT) were analyzed.

**Results:**

Among the 79 patients with incidental colorectal FDG uptake, histopathology revealed malignancy in 22 (27.9%) patients and premalignant polyps in 22 (27.9%) patients. Compared to patients with benign lesions, patients with premalignant and malignant lesions were more likely to undergo a PET/CT scan for primary evaluation (p = 0.013), and more likely to have focal GIT uptake (p = 0.001) and CWT (p = 0.001). A ROC curve analysis was made and assesed a cut-off value of 7.66 SUVmax (sensitivity: 64.9% and specificity: 82.4%) to distinguish premalignant and malignant lesions from benign lesions. The AUCs of the SUVmax and the combined parameters of SUVmax and CWT were 0.758 and 0.832 respectively.

**Conclusion:**

For patients undergo PET/CT for primary evaluation, imaging features of colorectal focal FDG uptake and CWT were more closely associated with premalignant and malignant lesions. The SUVmax helps determine benign and premalignant/malignant lesions of the colorectum. Moreover, the combination of SUVmax and CWT parameters have higher accuracy in estimating premalignant and malignant lesions than SUVmax.

## Introduction

Colorectal cancer (CRC) is the second leading cause of cancer death and the third most frequently diagnosed cancer worldwide (Bray et al. 2018). It is worth noting that 95% of colorectal cancer mainly comes from adenoma polyps (Aarons et al. 2014). Conventional adenomas (tubular, fluff and tubular) and jagged polyps are the main types of pre-malignant lesions. Early identification and treatment of colorectal premalignant and malignant lesions can greatly enhance survival rates.

The uses of Positron emission tomography/computed tomography (PET/CT) in clinical practice for CRC include diagnosing tumors, staging disease, restaging disease, and evaluating treatment efficacy(Bulens et al. 2018; Rodríguez-Fraile et al. 2020). As a result of increased PET/CT utilization in the cancer patients and the elderly population, the incidental detection of colorectal premalignant and malignant lesions has increased (Adams et al. 2018). However, the uptake of ^18^F FDG in the colorectum displays significant variability, with levels ranging from mild to intense and presenting in focal, diffuse, or segmental distributions. While diffuse or segmental patterns of FDG uptake typically stem from physiological or inflammatory processes, studies have indicated that 40.6–66.2% of focal incidental colorectal FDG uptake cases are associated with advanced colorectal neoplasia (Minamimoto et al. 2014; Seivert et al. 2014). These findings present diagnostic and therapeutic challenges for clinicians.

It is reported that a strong correlation exists between the site of FDG uptake and the presence of lesions as observed during colonoscopy (Prabhakar et al. 2007). For this reason, several studies have recommended thorough evaluation of all incidentally detected colonic foci through colonoscopy (Kei et al. 2010; Putora et al. 2013). However, colonoscopy is an invasive procedure that carries the risk of complications such as intestinal perforation, bleeding, anesthesia-related risks, and the need for meticulous bowel preparation (Bielawska et al. 2018; Kim et al. 2019) (Latos et al. 2022). The decision on whether to proceed with further colonoscopy evaluations for patients with incidental colorectal ^18^F-FDG uptake during PET/CT exams remains a topic of debate (Mainenti et al. 2011).

Our main objective of this study was to determine investigate the patterns and degrees of incidental colorectal uptake that are most likely to be associated with pre-malignant and malignant lesions in patients undergoing whole-body PET examination.

## Materials and methods

### Patient selection

The retrospective review of medical records was conducted on patients who underwent PET/CT scans at The First Affiliated Hospital of Soochow University from December 2019 to December 2023. This study was approved by the ethics committee of The First Affiliated Hospital of Soochow University (Approval number 2024169). Informed consent was waived for this retrospective study, and no personal information was disclosed. This study was in accordance with the Declaration of Helsinki. An incidental finding was characterized by an unexpected area of elevated FDG uptake in the ascending colon, transverse colon, descending colon, sigmoid colon, or rectum. Focal incidental uptake was identified when the radiology report described a focal pattern, while diffuse uptake was defined as increased FDG uptake along a continuous segment of the colorectum. A total of 79 eligible patients who had undergone colonoscopic examination and exhibited incidental colorectal FDG uptake were included in the study, excluding those with FDG uptake in regions of known colorectal malignancy.

The data collected comprised variables such as age, gender, PET indication, primary malignancy type, colonic wall thickening (CWT), location of incidental finding, FDG uptake pattern, and endoscopic and histopathological diagnoses. Histopathological findings were categorized as malignant (intramucosal or invasive adenocarcinoma), premalignant adenomatous polyps (including tubular, villous, and tubulovillous), and non-neoplastic/negative results. Approval for the study was obtained from The First Affiliated Hospital of Soochow University.

### ^18^F-FDG PET/CT imaging

All the FDG PET/CT images were obtained from the US GE Discovery STE 16 PET/CT scanner. The patients were fasted for at least 6 h prior to the intravenous injection of ^18^F-FDG (4.07–5.55 M Bq/kg). Patients’ blood glucose levels were checked just before the injection of FDG. Blood.

glucose level had to be < 11 mmol/L before injection in all patients. After intravenous injection of ^18^F-FDG for an average of 60 ± 10 min, imaging data were obtained using low-dose CT (140 kV, 120 mA, transaxial FOV 70 cm, pitch 1.75, rotation time 0.8 s, slice thickness 3.75 mm), followed.

By PET emission images, 2–3 min per bed position. The acquired data were reconstructed using an iterative algorithm in transverse, coronal, and sagittal planes and transferred to Advantage Windows Workstation (Advantage Windows Server 4.5; GE Healthcare) for processing and interpretation. The PET and coregistered PET/CT images were interpreted both visually and semiquantitatively by 10-year and 24-year experienced two nuclear medicine physicians. The maximum standardized uptake value (SUVmax), representing the FDG uptake degree, was measured by drawing a region of interest from the trans axial slice with the highest uptake of FDG for each abnormal colonic FDG uptake site in the attenuation-corrected PET data. Besides, ≥ 3 mm for the colon and ≥ 5 mm for the rectum were considered as increased wall thickness.

### Colonoscopy

Colonoscopies were performed by endoscopists from the Endoscopy Department of the First Affiliated Hospital of Soochow University. 79 patients received standard bowel preparation and underwent total colonoscopy from the anal verge to the cecal pole. The time of colonoscopy, macroscopic characteristics, location size, and number of lesions were recorded. At the time of colonoscopy, biopsy or polypectomy were performed on abnormal polypoidal and flat lesions. All specimens underwent histopathological analysis.

### Data analysis and statistical analysis

Statistical analysis was performed using SPSS software 23.0, (Inc.). The statistical analysis consisted of descriptive and inferential statistics. Mean (x) and standard deviation (SD) were used in the analysis of continuous data. Frequency (n) and percentage values (%) were used when defining categorical variables. Signifcant diferences of variable characteristics between groups were compared by Student’s t-test for the continuous variables, and χ2 tests for the categorical variables. A receiver operating characteristic (ROC) curve was plotted to define the optimal cut-off point to differentiate premalignant and malignant lesions from benign ones. The area under the curve (AUC) of SUVmax and the combined parameters of SUVmax and CWT were calculated. The results included hazard ratios and 95% confidence intervals (CIs). The statistical significance level of the data was taken as < 0.05.

## Results

### 3.1.Patient characteristics

**Fig. 1:**
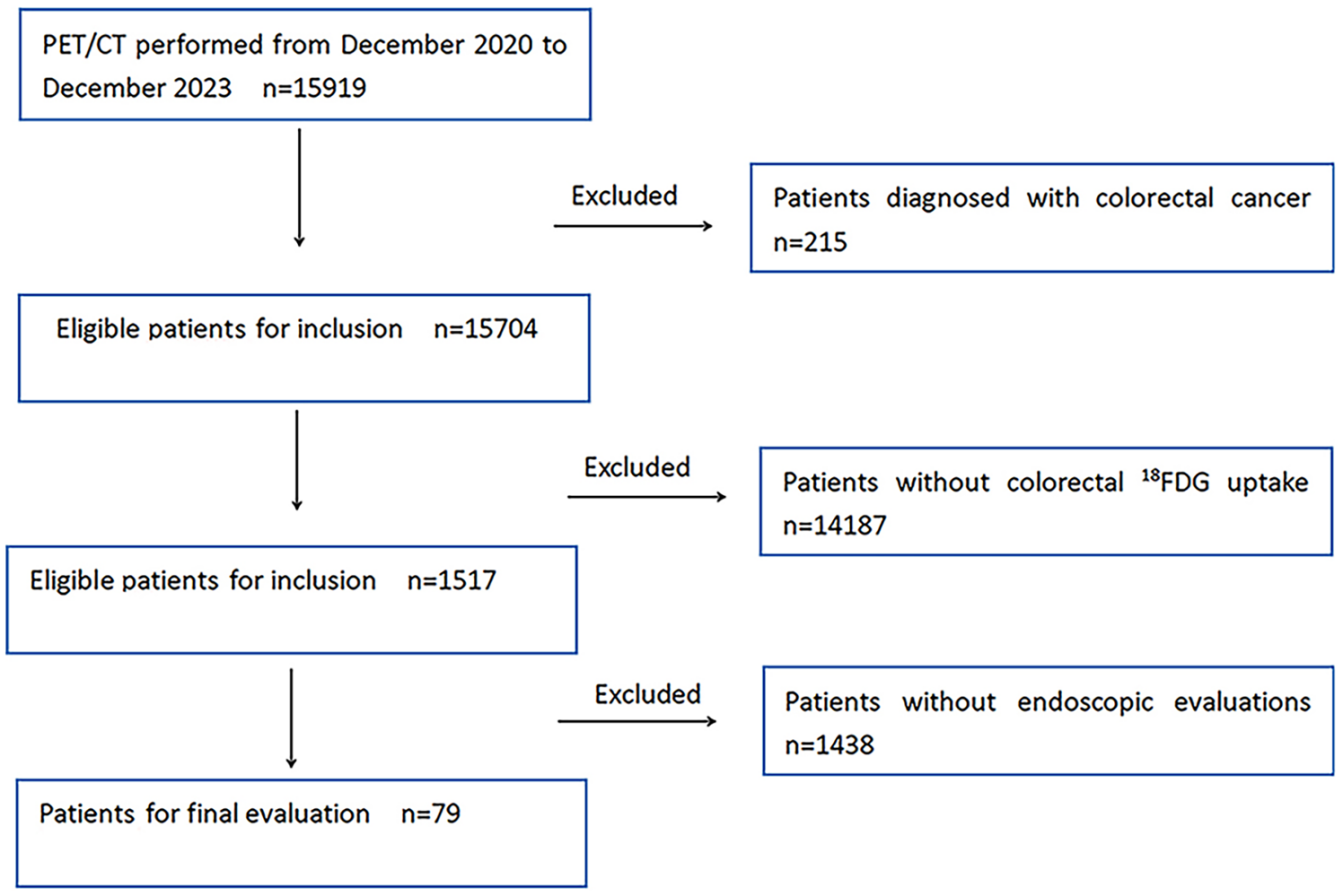
Flow diagram of patient recruitment

Out of 15919 FDG-PET/CT scans conducted, 1517 individuals exhibited incidental FDG uptake on PET/CT and lacked a prior history of colorectal cancer. 1438 participants were disqualified due to the absence of endoscopic assessments. Ultimately, the study enrolled 79 subjects (37 women, 42 men). Refer to Fig. 1 for the study's flowchart.

The characteristics and indications for FDG PET/CT in 79 patients were detailed in Table 1, showing a mean age of 62.92 ± 12.1 years. The distribution of uptake patterns revealed focal uptake in 31.6% of cases and diffuse uptake in 68.4% of cases. Among the colorectal FDG uptake occurrences, the distribution was as follows: 16 in the ascending colon, 4 in the transverse colon, 11 in the descending colon, 29 in the sigmoid colon, 2 in the rectum, and 17 in multiple colorectal locations. Of the 79 lesions detected by ^18^F-FDG PET/CT, 50.6% displayed CWT during the scan, while 49.4% did not. The primary indication for PET/CT was the initial evaluation of cancer, accounting for 58.2% of cases. The most prevalent types of primary malignancies identified were as follows: 24.1% lung carcinoma, 7.6% gastric carcinoma, 7.6% hepatoma, and 6.3% non-Hodgkin’s lymphoma lesions.

**Table 1:** General characteristics of 79 patients. Other* includes cervical cancer, diabetes, breast cancer, unexplained fever, unexplained abdominal pain, multiple myeloma, ovarian cancer, rheumatoid arthritis, sarcoidosis and rheumatic heart disease

General characteristics		Number(*n* = 79)	Percentage(%)
Gender	Male	42	53.2
	Female	37	46.8
Indication for PET/CT	Primary evaluation/staging	46	58.2
	Follow-up	33	41.7
Type of Primary	Lung carcinoma	19	24.1
	Gastric carcinoma	6	7.6
	Hepatoma	6	7.6
	Non-Hodgkin’s Lymphoma	5	6.3
	Renal carcinoma	4	5.1
	Bone carcinoma	4	5.1
	Other*	35	44.2
FDG Uptake	Focal	25	31.6
	Diffuse	54	68.4
Location of Uptake	Ascending colon	16	20.3
	Transverse colon	4	5.1
	Descending colon	11	13.9
	Sigmoid colon	29	36.7
	Rectum	2	2.5
	Mixed	17	21.5
Colonic wall thickening	Absent	39	49.4
	Present	40	50.6

### Endoscopy and histopathology results

Among 79 patients who underwent colonoscopy, 11 patients (13.9%) showed no abnormalities, while the remaining 68 patients (86.1%) had positive endoscopic results. Specifically, 8 patients (10.1%) were found to have benign adenomatous polyps, while 22 patients (27.8%) displayed pre-malignant lesions characterized by a potential for malignant progression. The most commonly identified pre-malignant lesion was tubulovillous adenoma, present in 8 out of the 22 cases (36.3%), followed by tubular adenoma in 7 cases (31.8%), high-grade intraepithelial neoplasia in 6 cases (27.2%), and villous adenoma in 1 case (4.5%), as outlined in Table 2.

**Table 2:** Pre-malignant and malignant lesions found upon endoscopic investigation

Pre-malignant polyps (22)	*n*(%)
Tubular adenoma	7(31.8)
Tubulovillous adenoma	8(36.3)
Villous adenoma	1(4.5)
High grade intraepithelial neoplasia	6(27.2)
Malignant lesions (*n* = 22)	n(%)
Moderately differentiated adenocarcinoma	13(59.1)
Poorly differentiated adenocarcinoma	5(22.7)
Lymphoma	1(4.5)
Ovarian metastatic tumor	1(4.5)
Neuroendocrine tumor	1(4.5)
Gastric metastatic tumor	1(4.5)

**Fig. 2:**
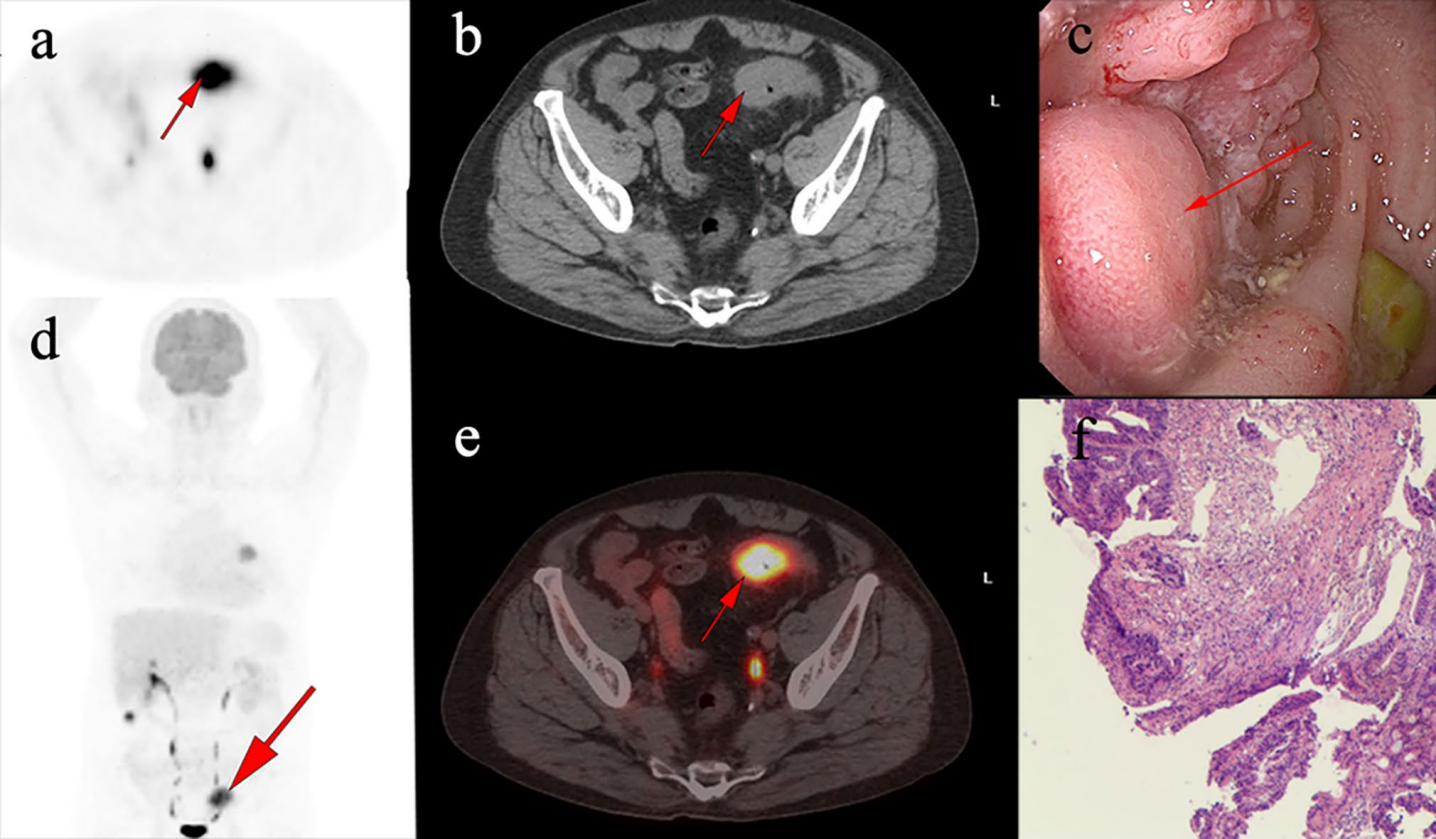
A 69-year-old man underwent ^18^F-FDG PET/CT for left lung carcinoma. Incidental focal ^18^F-FDG uptake was found in the sigmoid colon. The patient underwent colonoscopy and pathology examination. **a-b**, **d-e** Abnormal ^18^F-FDG PET/CT uptake in the sigmoid colon. **c** shows a sigmoid colon polyp with a diameter of 3.4 cm. **f** shows the pathological pattern of high-grade intraepithelial neoplasia

**Fig. 3:**
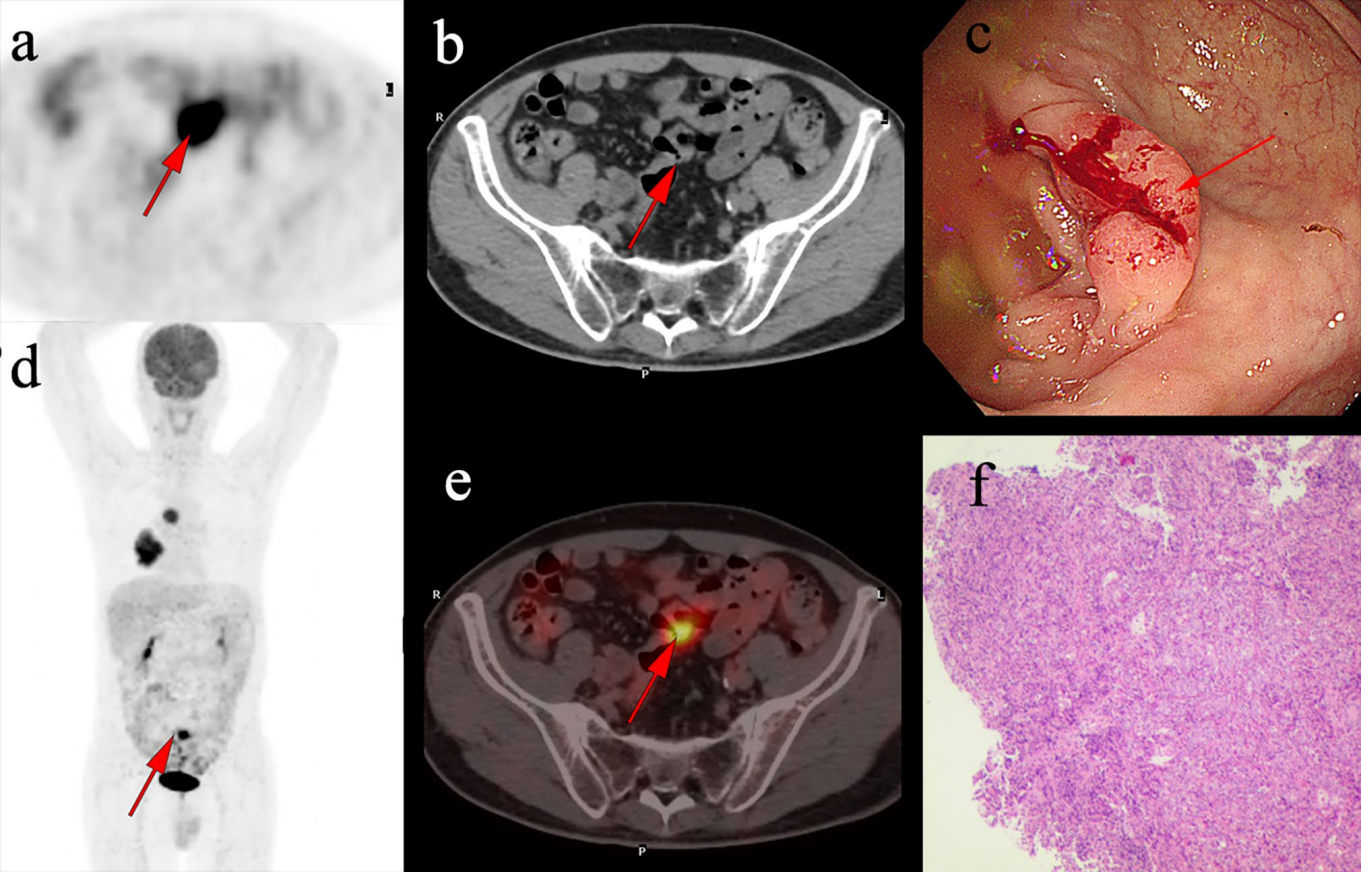
A 62-year-old man underwent ^18^F-FDG PET/CT for right lung carcinoma. Incidental focal ^18^F-FDG uptake was found in the sigmoid colon. The patient underwent colonoscopy and pathology examination. **a-b**, **d-e** Abnormal ^18^F-FDG PET/CT uptake in the sigmoid colon. **c** shows a sigmoid colon polyp with a diameter of 2.3 cm. **f** shows the pathological pattern of poorly differentiated adenocarcinoma

The characteristics of identified pre-cancerous lesions can be found in Tables 3. Within this cohort, the mean age of the patients was 63.86 ± 9.2 years, with 18 (81.7%) individuals displaying focal FDG uptake, and 12 (54.5%) showing pre-cancerous lesions in the sigmoid colon. A total of 13 (59.1%) lesions exhibited CWT during ^18^F-FDG PET/CT imaging, while 9 (40.9%) did not. The primary reason for undergoing PET/CT scans was for initial cancer assessment or staging (15, 68.2%). An exemplar of pre-cancerous lesions is illustrated in Fig. 2. Malignant lesions were identified in 27.8% (n = 22) of patients (Tables 4). The majority of these patients exhibited focal involvement (19, 86.4%), while diffuse involvement was noted in 13.6% (n = 3) of cases. Among these cases, 13 were situated in the sigmoid colon, 4 in the ascending colon, and the remaining 4 in the descending colon and rectum, respectively. Consistent with the pre-cancerous group, the primary indication for PET/CT scans was for the initial evaluation or staging of cancer (16, 72.7%). CWT was observed in 77.3% (n = 17) of malignant lesions during ^18^F-FDG PET/CT imaging. An example is provided in Fig. 3.

**Table 3:** Clinical characteristics ofincidental colorectal FDG uptake of pre-cancer lesions

Characteristics		Incidental intestinal tract pre-malignant (*n* = 22)
Gender, n (%)	Male	13(59.1)
	Female	9(40.9)
Age, mean ± SD		63.86 ± 9.2
Location of Uptake	Ascending colon	3(13.6)
	Transverse colon	1(4.5)
	Descending colon	2(9.1)
	Sigmoid colon	12(54.5)
	Rectum	1(4.5)
	Mixed	3(13.6)
Colonic wall thickening	Absent	9(40.9)
	Present	13(59.1)
Indication	Primary evaluation/staging	15(68.2)
	Follow-up	7(31.8)
FDG uptake, *n* (%)	Focal	18(81.7)
	Diffuse	4(18.2)
SUVmax, mean ± SD		9.89 ± 6.7

**Table 4:** Clinical characteristics ofincidental colorectal FDG uptake of cancer lesions

Characteristics		Incidental intestinal tract malignancy (*n* = 22)
Gender, *n* (%)	Male	8(36.4)
	Female	14(63.6)
Age, mean ± SD		65.23 ± 13.1
Location of Uptake	Ascending colon	4(18.2)
	Transverse colon	0(0)
	Descending colon	3(13.6)
	Sigmoid colon	13(59.1)
	Rectum	1(4.5)
	Mixed	1(4.5)
Colonic wall thickening	Absent	5(22.7)
	Present	17(77.3)
Indication	Primary evaluation/staging	16(72.7)
	Follow-up	6(27.3)
FDG uptake, *n* (%)	Focal	19(86.4)
	Diffuse	3(13.6)
SUVmax, mean ± SD		10.78 ± 5.9

### Statistical analysis

**Fig. 4:**
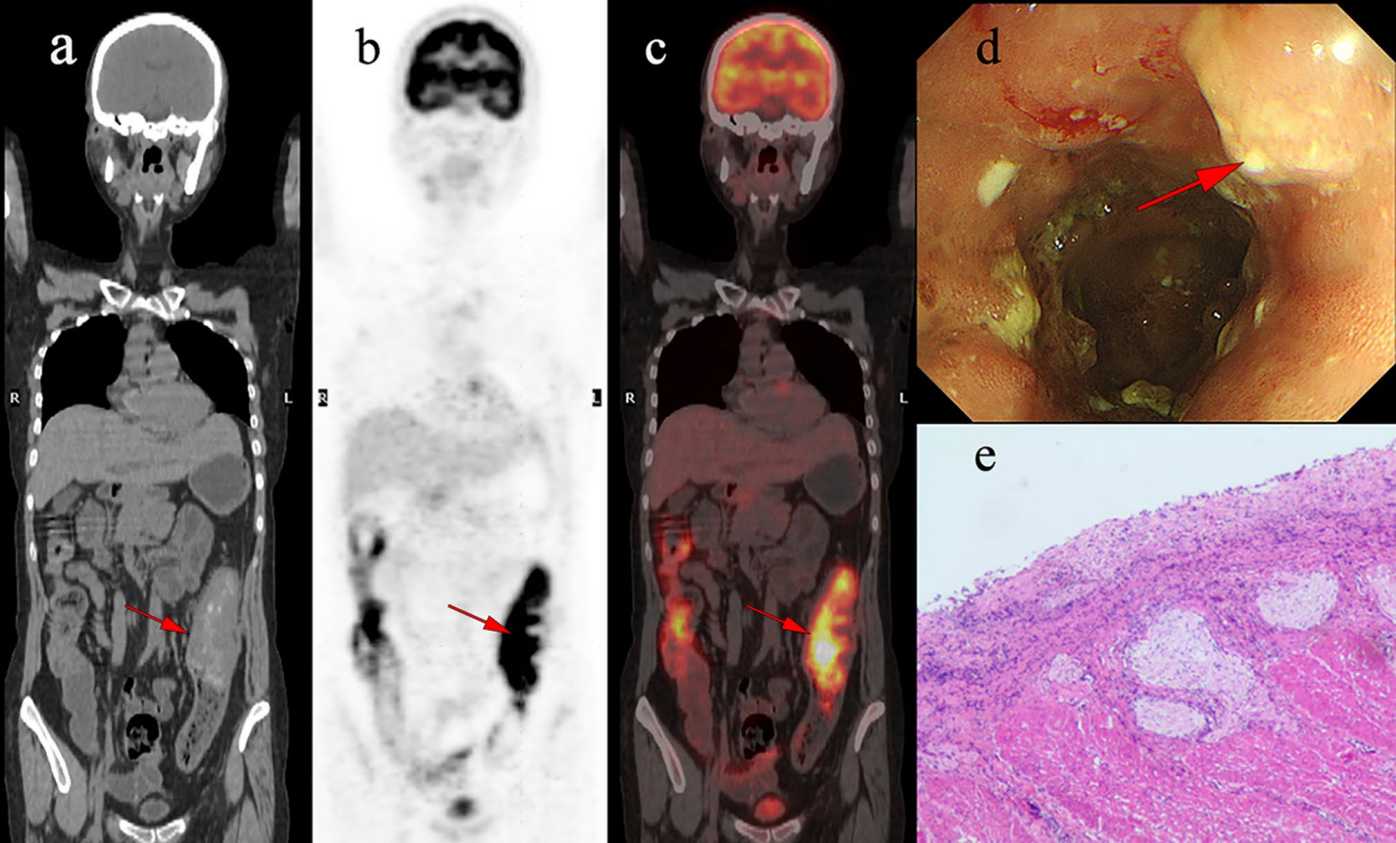
A 45-year-old woman underwent ^18^F-FDG PET/CT for unexplained abdominal pain. Incidental diffuse ^18^F-FDG uptake was found in the colon. The patient underwent colonoscopy and pathology examination. **a-c** Abnormal ^18^F-FDG PET/CT uptake in the colon. **d** shows diffuse thickening of the colon wall. **e** shows the pathological pattern of Crohn’s disease.

**Fig. 5:**
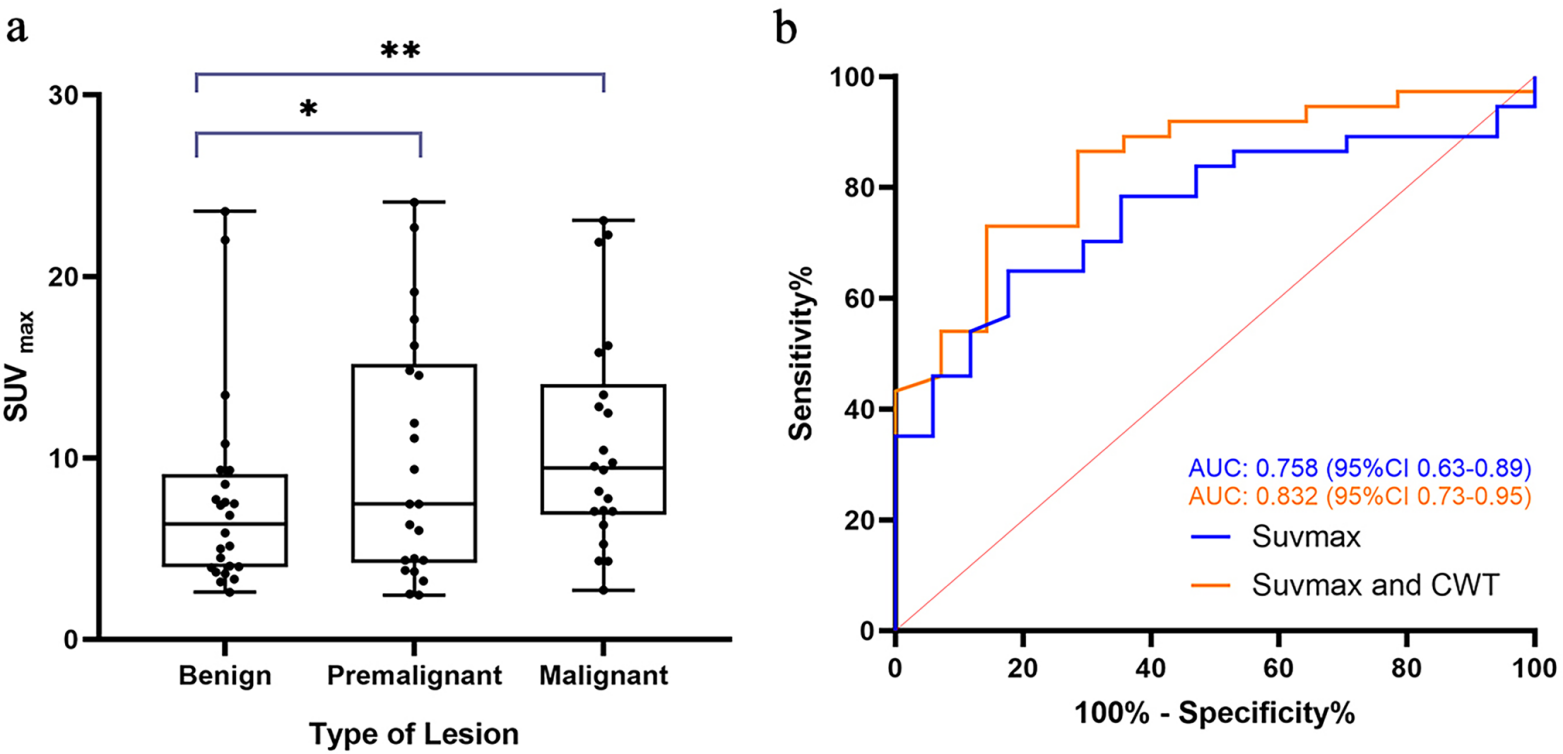
**a** SUVmax of benign, pre-malignant, malignant lesions were compared; **b** ROC curve of maximum standardized uptake value (SUVmax) and the combined parameters of SUVmax and CWT for pre-malignant/malignant lesions

The characteristics of identified pre-cancerous lesions are outlined in Tables 3. Within this cohort, Patients' average ages were segregated into two groups and compared based on histopathological findings (Tables 5). No statistically significant variances were discovered between groups in terms of age and gender. Nevertheless, there exists a significant statistical contrast in the uptake pattern, CWT, and PET/CT scan indication between the groups (p < 0.05). Upon contrasting the SUVmax values among three distinct categories (malignant, pre-malignant, and benign lesions), it is evident that the pre-malignant and malignant groups display notable variations from the benign group (p < 0.05) (Fig. 5a), with an example of a benign discovery depicted in Fig. 4. ROC curve analysis was conducted to assess the precision of SUVmax and the concatenated parameters of SUVmax and CWT in distinguishing pre-malignant and malignant lesions from benign ones. The optimal SUVmax cut-off was determined to be 7.66 (sensitivity 64.9%, specificity 82.4%), marking lesions with SUVmax exceeding ≥ 7.66 as bearing high risks of pre-malignant or malignant lesions (Fig. 5b). The AUC values for SUVmax and the combined SUVmax and CWT parameters were 0.758 and 0.832, respectively. The accuracy and sensitivity of diagnosing pre-malignant/malignant lesions were enhanced when lesions displaying SUVmax ≤ 7.66 were combined with CWT parameters (Table 6).

**Table 5:** comparing the clinical characteristics of patients with benign and malignant findings upon colonoscopy evaluation

Variable	Cancer/pre-cancer lesions (44)	Benign findings (24)	*p*-Value
Age (mean ± SD)	64.55 ± 11.4	60.89 ± 12.6	0.122
Gender (male)	21(47.7%)	16(66.6%)	0.219
Focal uptake	37(48.6%)	12(50.0%)	0.001
Colonic wall thickening	30(68.1%)	8(33.3%)	0.001
Indication (primary evaluation)	31(70.4%)	21(87.5%)	0.013
SUVmax (mean ± SD)	11.45 ± 6.3	6.28 ± 2.9	0.02

**Table 6:** Sensitivity and specificity of SUVmax and SUVmax combined with CWT in the identification of pre-malignant/malignant lesions from incidental colorectal ^18^F-FDG uptake foci

Measures(SUVmax)	≥ 4	≥ 5	≥ 6	≥ 7	≥ 7.66	≥ 8	≥ 9	≥ 10	≥ 11
Sensitivity(%)	0.892	0.865	0.811	0.73	0.649	0.622	0.568	0.486	0.459
Specificity(%)	0.235	0.471	0.529	0.647	0.824	0.824	0.824	0.882	0.941
Sensitivity(%) combined with CWT	0.941	0.9	0.867	0.767	0.667	0.623	0.567	0.467	0.433
Specificity(%) combined with CWT	0.335	0.547	0.587	0.655	0.828	0.828	0.828	0.911	0.921

## Discussion

PET/CT is a non-invasive technique for diagnosing, staging, monitoring treatment response, assessing tumor aggressiveness, and determining radiotherapy areas (Tatsumi et al. 2007). Incidental or unexpected FDG uptake in the colon or rectum is not rare (Jati et al. 2012; şimşek et al. 2015), Our study is one of the largest to include any incidental colorectal uptake that was followed-up endoscopically. The rate of incidental colorectal uptake in our study population was 9.6%, with 79 patients (5.2%) undergoing further investigation. A meta-analysis reported a pooled prevalence of incidental colorectal FDG uptake in FDG-PET or PET/CT to be 3.1–3.6% (Treglia et al. 2014; Elzaki et al. 2022). Our study shows a higher rate of FDG uptake in the colorectum than found in previous studies. However, these studies only included local uptake, while our study included diffuse uptake in the colon and rectum. A partial explanation lies in the advances in the technology and equipment combined with a higher level of interpretation expertise. In our study, 68 patients had abnormal findings, including benign, malignant and premalignant lesions and gave us a positive predictive value of 86.1%, which was comparably higher than the 47–63% detected in similar studies (Kousgaard et al. 2020; Albertsen et al. 2022).

Adenomas were divided into two categories benign and pre-malignant adenomas according to the guideline prepared by the European Society of Gastrointestinal Endoscopy (Hassan et al. 2013). Our study, confirmed by colonoscopy and pathology, suggested an equal proportion of cancerous lesions and premalignant adenomas in incidental ^18^F-FDG uptake (27.8%). However, there were still a considerable proportion of normal or inflammatory lesions and benign adenomas (44.3%). It is thought that the majority of colorectal cancers develop from adenomatous polyps, and early detection may prevent progressive malignant disease (Winawer et al. 2000; Click et al. 2018), Therefore, pre-malignant adenomas should also be treated promptly. In our study, the pre-malignant adenomas included 7 cases of tubular adenoma, 8 tubulovillous adenoma, 1 villous adenoma and 6 high grade intraepithelial neoplasia. This result is consistent with other studies showing that FDG PET/CT is a sensitive tool to detect colonic premalignant lesions (Winawer et al. 2000; Weston et al. 2010).

In clinical practice, it would be of benefit for the clinician to have a general idea of the probability of malignancy based on uptake pattern and location. However, FDG may physiologically accumulate in the gastrointestinal tract in focal, segmental, or diffuse form. The etiology of this physiological uptake by the colon is multifactorial and quite variable, which can be related to smooth muscle activity, mucosal and mucosal-associated lymphoid tissue activity, microbial activity, swallowed secretions, and active FDG excretion (Jayaprakasam et al. 2021), Several studies have investigated that diffuse segmental FDG uptake in the colon is mainly observed physiologically, intense focal uptake is more often associated with actual lesions (Son and Kim 2019; Kirchner et al. 2020). Our findings illustrated a strong correlation between incidental focal FDG uptake on PET/CT and abnormal lesion. The most common lesion site is the sigmoid colon in the cancer/pre-cancer group. It is worth noting that 15.9% of all patients with an abnormal lesion on endoscopy had diffuse FDG uptake on PET, which was consistent with the 1–17% detected in similar studies (Lu et al. 2013).

Our study showed that the intensity of the uptake was an independent predictor of finding a cancer/pre-cancer lesion. Through ROC curve analysis, a cut-off value of SUVmax was established at 7.66, with a sensitivity of 64.9% and specificity of 82.4%. Ahmet Cem Esmer et al. (Esmer et al. 2023). defined a cut-off point of 11.1 SUVmax (ensitivity 83.3%, specificity 90%) with a ROC curve to distinguish benignity from malignancy. Mohammad N. Hosni et al. (Hosni et al. 2023) showed a sensitivity of 0.76 and a specificity of 0.885 at an SUVmax cut-off of 9.2 for the differentiation between benign and cancer/pre-cancerous lesions. However, some studies (Treglia et al.^ 2014^; şimşek et al.^ 2015^) concluded that SUVmax should not be used alone to differentiate between malignant, premalignant, and benign lesions. In order to increase the discrimination efficiency between benign and malignant lesions, our study evaluated SUVmax and localized CWT parameters and found that the AUCs of the SUVmax and the combined parameters of SUVmax and CWT were 0.758 and 0.832 respectively, which indicated that the combined parameters have higher accuracy in estimating precancerous or malignant lesions than SUVmax.

CWT is important for the detection of neoplastic lesions of the colorectum. Premalignant/malignant lesion rate is reported to be 15%-65% in CWT studies (Chandrapalan et al. 2018). A prospective study by Khairnar et al. observed a cancer rate of 11.7% in CWT patients and showed that irregular or moderate-severe wall thickening can predict cancer (Khairnar et al. 2019). However, this study evaluated the group of patients with CWT incidentally diagnosed via CT. With the advent of PET/CT hybrid imaging, many studies have focused on the effects of FDG uptake and wall thickening on CT on diagnostic performance. In Ahmet Cem Esmer et al. study (Esmer et al. 2023), of 17 patients with segmental FDG uptake on PET/CT and wall thickening on CT, malignancy was detected in seven, polyps were detected in one, and a negative result was observed in the remaining nine patients. However, a deficiency of this study is that there is no quantitative analysis on the diagnostic effectiveness of premalignant/malignant lesion. Wenmin Xu et al. (Xu et al. 2022) Showed CWT was used as the evaluation parameter of benign and malignant lesions of incidental focal colorectal FDG uptake to improve the specificity of lesions with SUVmax < 6.45 by retrospectively analysing 44 colonoscopy reports, including 37 patients with CWT. In contrast, in our study, when SUVmax ≤ 7.66, the combination of CWT parameters can improve the specificity and the sensitivity of detection of premalignant/malignant lesion lesions. Whereas their study included incidental focal colorectal FDG uptake, ours included both focal and diffuse FDG uptake. In our study, Among 4 patients with diffuse FDG uptake on PET/CT and wall thickening on CT, 2 had chronic colitis, 1 was Crohn's disease and a negative result was observed in one patient.

The retrospective nature of the study is the first limitation. Other limitations are that our sample size was relatively limited, and either a standard bowel preparation regimen or intravenous contrast material, which is suggested for better image recognition, was not applied due to the study’s retrospective nature. In addition, we observe that FDG uptake occurs in both benign and malignant colorectal lesions (Fig. 5a). Therefore, the diagnostic properties of FDG for benign and malignant colorectal lesions were limited. It is necessary to develop new molecular probes to identify benign and malignant colorectal lesions. To this end, some research teams proposed ^68^ Ga-labeled fibroblast activation protein inhibitor-04 (^68^ Ga-FAPI-04) (Li et al. 2024) and ^89^Zr-atezolizumab (anti-PD-L1) (Bensch et al. 2018) PET imaging has shown promising value in colorectal cancer detection. Moreover, the ^64^Cu-DOTA-cetuximab-F(ab’)_2_ molecular probe has achieved remarkable results in identifying colon cancer and colitis, although it has only been verified in animal models (Turker et al. 2014).

## Conclusions

In conclusion, our study highlights that in patients with incidental colorectal FDG uptake, imaging findings of focal uptake and CWT were more closely associated with premalignant and malignant lesions. An established SUVmax cut-off of 7.66 demonstrated robust sensitivity and specificity for differentiating premalignant and malignant lesions from benign lesions. Moreover, the combination of SUVmax and CWT parameters have higher accuracy in estimating precancerous or malignant lesions than SUVmax. These findings contribute to our understanding of the clinical significance of incidental colonic uptake of FDG and highlight the importance of follow-up colonoscopy for further evaluation.

## Data Availability

The patient data used to support the fndings of this study are available from the corresponding author upon request.
